# *Fusarium graminearum* Ste3 G-Protein Coupled Receptor: A Mediator of Hyphal Chemotropism and Pathogenesis

**DOI:** 10.1128/msphere.00456-22

**Published:** 2022-11-15

**Authors:** Tanya Sharma, Pooja S. Sridhar, Christopher Blackman, Simon J. Foote, John S. Allingham, Rajagopal Subramaniam, Michele C. Loewen

**Affiliations:** a Department of Chemistry and Biomolecular Sciences, University of Ottawa, Ottawa, Ontario, Canada; b Aquatic and Crop Resources Development Research Center, National Research Council of Canada, Ottawa, Ontario, Canada; c Department of Biomedical and Molecular Science, Queen’s University, Kingston, Ontario, Canada; d Department of Cell and Systems Biology, University of Toronto, Toronto, Ontario, Canada; e Agriculture and Agri-Food Canada, Ottawa, Ontario, Canada; f Human Health Therapeutics Research Center, National Research Council of Canada, Ottawa, Ontario, Canada; University of Georgia

**Keywords:** hyphal chemotropism, *Fusarium graminearum*, Fusarium head blight, G-protein coupled receptors, wheat infection, pheromone receptor, wheat disease

## Abstract

Fungal hyphal chemotropism has been shown to be a major contributor to host-pathogen interactions. Previous studies on Fusarium species have highlighted the involvement of the Ste2 G-protein-coupled receptor (GPCR) in mediating polarized hyphal growth toward host-released peroxidase. Here, the role of the opposite mating type GPCR, Ste3, is characterized with respect to Fusarium graminearum chemotropism and pathogenicity. *Fgste3Δ* deletion strains were found to be compromised in the chemotropic response toward peroxidase, development of lesions on germinating wheat, and infection of Arabidopsis thaliana leaves. In the absence of *Fg*Ste3 or *Fg*Ste2, F. graminearum cells exposed to peroxidase showed no phosphorylation of the cell-wall integrity, mitogen-activated protein kinase pathway component Mgv1. In addition, transcriptomic gene expression profiling yielded a list of genes involved in cellular reorganization, cell wall remodeling, and infection-mediated responses that were differentially modulated by peroxidase when *Fg*Ste3 was present. Deletion of *Fg*Ste3 yielded the downregulation of genes associated with mycotoxin biosynthesis and appressorium development, compared to the wild-type strain, both in the presence of peroxidase. Together, these findings contribute to our understanding of the mechanism underlying fungal chemotropism and pathogenesis while raising the novel hypothesis that *Fg*Ste2 and *Fg*Ste3 are interdependent on each other for the mediation of the redirection of hyphal growth in response to host-derived peroxidase.

**IMPORTANCE** Fusarium head blight of wheat, caused by the filamentous fungus Fusarium graminearum, leads to devastating global food shortages and economic losses. Fungal hyphal chemotropism has been shown to be a major contributor to host-pathogen interactions. Here, the role of the opposite mating type GPCR, Ste3, is characterized with respect to F. graminearum chemotropism and pathogenicity. These findings contribute to our understanding of the mechanisms underlying fungal chemotropism and pathogenesis.

## INTRODUCTION

In nature, fungi exist as sessile organisms that conduct spatiotemporal sensing and associated responses through their hyphae. Thus, their abilities to extend and control the trajectory of hyphal extension is tightly coordinated. A range of environmental cues control this hyphal behavior by either acting as a positive stimulus, encouraging hyphal growth, as seen in the case of nutrients, mating pheromones, and host signals, or acting as a negative stimulus, repelling the hyphae away as in the case of toxins (1). The phenomenon of polarized hyphal growth toward or away from external stimuli is referred to as chemotropism, and is quintessential to fungal symbiotic, parasitic, and host-pathogen interactions.

A classic example of fungal chemotropism lies in the process of mating between *MATa* and *MATα* cell types in model organisms, such as Saccharomyces cerevisiae and Neurospora crassa, which highlights growth toward a pheromone gradient (2–9). Similarly, the chemotropic responses of hyphae to nutrient sources were also demonstrated in several saprophytic and parasitic oomycetes fungi (10–12). However, evidence of hyphal chemotropism in phytopathogenic fungi is relatively sparse. Some of the earliest reports were in the phytopathogen Cochliobolus sativus, which exhibited preferential growth toward barley roots (13). Others demonstrated that when located adjacent to their host roots, Phytophthora cinnamoni cysts germinated rapidly and grew in the direction of the roots (14). More recently, researchers showed that soilborne Fusarium oxysporum displayed positive chemotropic growth toward catalytically active class III peroxidases that were released by host tomato roots (15).

With respect to the molecular machinery mediating hyphal chemotropism, G-protein coupled receptors (GPCRs) have been shown to play a critical role (16–18). Characterized by their cell membrane localization and seven transmembrane domain structure, GPCRs classically function as molecular switches at which the binding of an external ligand elicits conformational changes in the receptor that lead to the dissociation of intracellular heterotrimeric G-proteins with a corresponding exchange of GDP to GTP on the G-α subunit (19). The released G-protein domains activate downstream signaling cascades, leading to the activation of various cellular responses. In the model system S. cerevisiae, the diffusible α-factor and a-factor pheromones bind their respective *Sc*Ste2p and *Sc*Ste3p receptors to mediate mating in a paracrine fashion (20, 21). The stimulation of these receptors activates the filamentous growth mitogen-activated protein kinase (MAPK) pathway (Ste11p, Ste7p, Fus3p, and Ste12p), thereby yielding physiological changes in each mating cell type and leading to cell cycle arrest and shmoo formation (3–9). A cooperative role for *Sc*Ste2p and *Sc*Ste3p in subsequent zygote development has also been proposed (2). In F. oxysporum, receptors *Fo*Ste2 and *Fo*Ste3 have been shown to be stimulated by pheromones in an autocrine fashion to control conidial germination in a density-dependent manner (22). High concentrations of the α-factor repress germination, but when Bar1 protease is secreted from the cells, it cleaves the α-factor, thereby increasing the relative concentration of the a-factor and relieving the repression.

Interestingly, Ste2 was recently shown to be responsible for perceiving and transmitting the peroxidase-mediated chemotropic responses in F. oxysporum, F. graminearum, and Verticillium dahliae, underscoring the general relevance of this GPCR in chemotropism (15, 23, 24). The deletion of Ste2 also led to a decrease in the virulence of these pathogens on their known hosts, including tomato roots, germinating wheat coleoptiles, and eggplant seedlings. The peroxidase-stimulated response in Fusarium and *Verticillium* species was shown to be transduced by the highly conserved cell wall integrity (CWI) MAPK pathway (15, 23, 24). Deletion mutants lacking genes integral to the CWI pathway, including Rho1, Bck1, Mkk2, and Mgv1 (Mpk1; Slt2) were impaired in peroxidase-mediated chemotropism. This is in contrast to genes belonging to the filamentous growth MAPK pathway, including Ste11, Ste7, Gpmk1 (Fmk1; Fus3), and Ste12, the deletion of which had no impact on peroxidase-mediated chemotropism. In addition to chemosensing, the CWI pathway has been implicated in pathogenesis by promoting infection and penetration through appressorium formation, the osmotic stress response, and deoxynivalenol (DON) biosynthesis (25–27).

The prior observation of synergistic relationships between Ste2 and its cognate pheromone receptor Ste3 raised the novel hypothesis that Ste3 may also play a role in host sensing. To address this, the chemotropic and virulence potential of *STE3* was explored through a reverse genetic approach, using a CRISPR-generated *FgSTE3* deletion mutant. Downstream signaling pathway activation and transcriptomic studies revealed the functional relevance and mechanistic aspects underlying the observed chemotropic responses to host peroxidase and associated pathogenesis.

## RESULTS

### Deletion of *F. graminearum STE3* compromises chemotropism toward peroxidase.

To determine whether *Fg*Ste3 is involved in mediating the chemotropic response to peroxidase, the annotated F. graminearum *STE3* gene sequence (*FGSG_07270* [28–30]) was deleted from F. graminearum. A CRISPR-CAS9-mediated transformation strategy was applied using a homology-directed repair mechanism, in which the entire *FgSTE3* open reading frame was replaced with a hygromycin-resistance gene cassette (Fig. S1). Hygromycin-resistant transformants were isolated (*Fgste3Δ-1*, *Fgste3Δ-2*, *Fgste3Δ-3*, and *Fgste3Δ-4*) and validated via polymerase chain reaction (PCR) amplification, using primers specific to the *FgSTE3* gene (Fig. S2). Sanger sequencing was performed on the obtained strains, and whole-genome sequencing was performed on strain *Fgste3Δ-3*, validating the insertion and confirming that the observed phenotype was exclusively due to the loss of *FgSTE3*, with no off-target effects.

10.1128/msphere.00456-22.1 FIG S1 Design of the protospacer regions for CRISPR-generated *Fgste3Δ* knockout. Sequences of the *STE3* upstream and downstream regions from the start and stop codons are shown. The PAM motifs are marked in red, and the start and stop codons are underlined in black. The sequences used for the microhomology regions are shown underlined in green, and the light blue color indicates the crRNA for both the 5′ and 3′ regions. Download FIG S1, TIF file, 0.8 MB. © Crown copyright 2022. 2022 Crown https://creativecommons.org/licenses/by/4.0/ This content is distributed under the terms of the Creative Commons Attribution 4.0 International license.

10.1128/msphere.00456-22.2 FIG S2 Confirmation of *STE3* deletion by PCR. The hygromycin-resistant transformants (*Fgste3Δ-1*, *Fgste3Δ-2, Fgste3Δ-3, Fgste3Δ-4*) were screened through PCR with the *STE3* ORF primers P3 and P4 (Table 1). The expected DNA fragment size is approximately equal to 1,400 bp. Download FIG S2, TIF file, 1.2 MB. © Crown copyright 2022. 2022 Crown https://creativecommons.org/licenses/by/4.0/ This content is distributed under the terms of the Creative Commons Attribution 4.0 International license.

**FIG 1:**
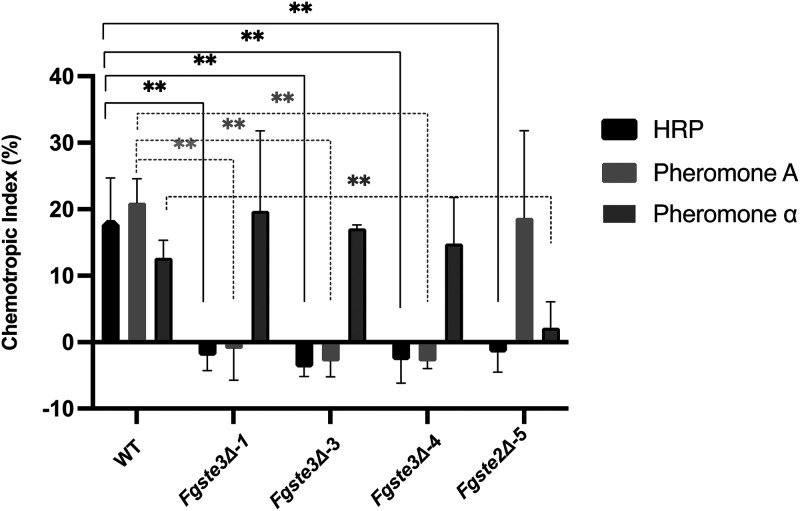
F. graminearum chemotropic growth toward peroxidase is mediated by both Ste3 and Ste2 receptors. Polarized hyphal growth of wild-type, *Fgste3Δ-1*, *Fgste3Δ-3*, *Fgste3Δ-4*, and *Fgste2Δ-5* were calculated 12 h after exposure to the indicated chemoattractants. Hyphae growing toward either 4 μM horseradish peroxidase (HRP), pheromones a-factor or α-factor (378 μM) were counted against a competing solvent control gradient (water or 50% vol/vol methanol, respectively). Data are representative of averages of 3 independent replicates (*n* = 500 hyphae/interaction/replicate; **, *P* < 0.001). Error bars represent the standard deviation. The statistics were assessed using Student’s *t* test.

The three positive *FgSTE3* deletion knockout strains were subjected to chemotropism plate assays and were screened against commercially available peroxidase (horseradish peroxidase [HRP]) (Fig. 1). The wild-type strain was assayed as a positive chemotropic control, and the previously characterized *Fgste2Δ-5* was also included for comparison and as a negative control (24). All three *Fgste3Δ* strains consistently showed random hyphal growth in the presence of HRP, compared to the directed growth observed for the wild-type strain. This indicates that the chemotropic response to HRP was completely abolished with the deletion of *FgSTE3*. Chemotropism of the wild-type strain was fully characterized again for the hyphal length, angle of hyphal growth, and sensitivity of the activity to active peroxidase, with the obtained values being consistent with those of its prior characterization in our previous work (Fig. S3A–C) (Sridhar et al., 2020 [24]).

10.1128/msphere.00456-22.3 FIG S3 Characterization of *Fgste3Δ-3* hyphal length and sensitivity to HRP activity. (A) Hyphal tip projection angle assay: Average cosine of hyphal tip projection angles were measured for the F. graminearum wild-type, *ΔFgste3-3*, and *ΔFgste2-5* strains towards a gradient of solvent control (C), horseradish peroxidase (HRP), Pheromone a (A) and Pheromone α (α). The data are presented as means from three experiments (*n* = 100 hyphae/interaction/replicate). Bars indicate the upper and lower 95% significance limits for the cosine means, according to a *t*-test. A cosine of 1 indicates a perfect orientation, while 0 indicates a random orientation. Chemotropism was considered significant when the lower confidence limit was >0. (B) Control experiment for the average length of the hyphae of the wild-type F. graminearum towards a gradient of solvent control (C) or horseradish peroxidase (HRP). The data are representative of three experiments with *n* = 100 hyphae. (C) The peroxidase chemoattractant sample was treated with its inhibitor, salicylhydroxamic acid (SHAM), at a concentration of 60 mM for 5 minutes, underwent heat denaturation at 95°C for 10 minutes, or was proteolysed by Proteinase K (1 mg/mL) for 30 minutes at room temperature. Download FIG S3, TIF file, 1.0 MB. © Crown copyright 2022. 2022 Crown https://creativecommons.org/licenses/by/4.0/ This content is distributed under the terms of the Creative Commons Attribution 4.0 International license.

### Deletion of *FgSTE2* or *FgSTE3* does not affect pheromone-induced chemotropism arising from the remaining opposite pheromone receptor.

The chemotropic preferences of *Fgste2Δ* or *Fgste3Δ* strains toward F. graminearum pheromones were also investigated. Hyphae growing toward either F. graminearum a-factor or α-factor pheromones were quantified compared to those of the wild-type (Fig. 1). While the wild-type showed a strong response toward both pheromones, the *Fgste3Δ* strains had no response to the a-factor, consistent with the absence of *Fg*Ste3. However, the chemotropic response to the α-factor was retained, consistent with the native expression of *FgSTE2* in these deletion strains. Similarly, the *Fgste2Δ-5* strain showed no chemotropic response to the *Fg*Ste2 α-factor pheromone, but it retained its response to the *Fg*Ste3 a-factor pheromone. Overall, the responses of these strains to pheromones differ from their responses to HRP, where all responses to HRP were lost, regardless of which receptor was deleted (Fig. 1). This finding suggests that different receptor mechanisms underlie the perception of the pheromones versus the HRP signal.

### *FgSTE3* deletion has no effect on cell wall stress responses or osmotic stress tolerance.

**FIG 2:**
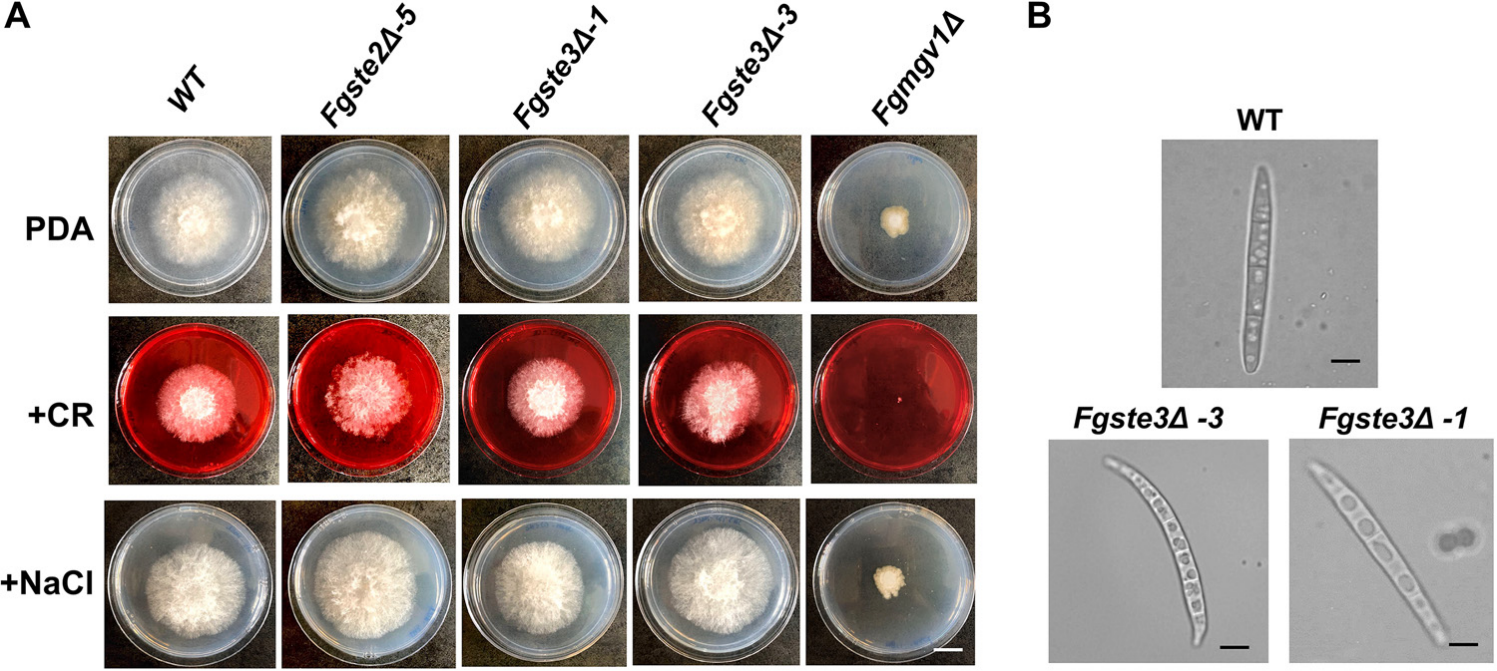
*FgSTE3* deletion has no effect on F. graminearum morphology or on osmotic stress tolerance. (A) Images of wild-type and mutant strains *Fgste2Δ-5*, *Fgste3Δ-1*, *Fgste3Δ-3*, and *Fgmgv1Δ* conidia grown on PDA, Congo red (CR; 150 μg/mL), and NaCl (0.7 M). Similar results were obtained for two independent experiments. Size bar = 1 cm. (B) Conidia for the wild-type, *Fgste3Δ-3* and *Fgste3Δ-1* were imaged under 100× using oil immersion. The images were captured using cellSens software, version 1.12. Size bar = 10 μM.

Initial observations of colony growth indicated no significant differences in the growth pattern, colony color, or morphology of the *Fgste3Δ* strains, compared to the wild-type strain or the *Fgste2Δ-5* strain. They all displayed normal growth with no visible change in the presence of Congo red (cell wall stressor) or NaCl (an osmotic stressor), respectively (Fig. 2A). Microscopic examination showed that the *Fgste3Δ-3* and *Fgste3Δ-1* conidia were slightly longer and narrower, compared to those of the wild-type (Fig. 2B).

### *FgSTE3* deletion leads to decreased virulence and pathogenicity.

**FIG 3:**
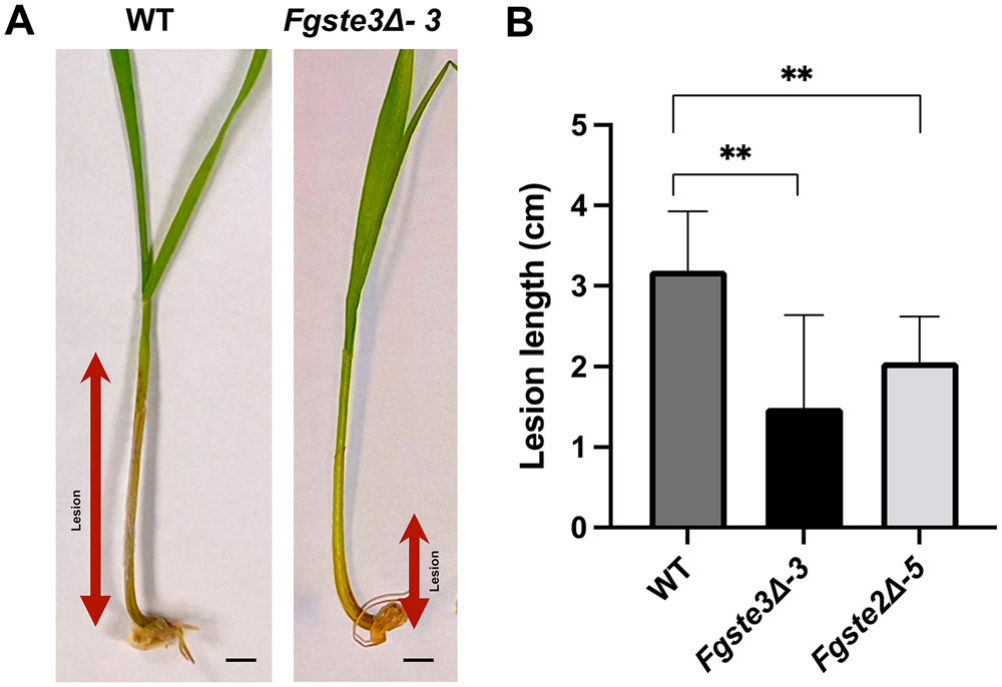
Deletion of *FgSTE3* leads to decreased F. graminearum pathogenicity against wheat. (A) The pathogenicity was quantified via the measurement of the length of infected stalk or lesion formed on germinating “Roblin” coleoptiles that were infected with the indicated F. graminearum strains. Shown are representations of lesions formed around the wound site 10 days after infection with F. graminearum conidia. (B) Quantification of average lesion length formed on germinating “Roblin” coleoptile stalks infected with F. graminearum wild-type and mutant strains *Fgste3Δ-3* and *Fgste2Δ-5.* The averages of two representative experiments are shown (compared to the wild-type strain; *n* = 18; **, *P < *0.005). Error bars represent the standard deviation. The statistics were assessed via Student’s *t* test. Size bar = 1 cm.

**FIG 4:**
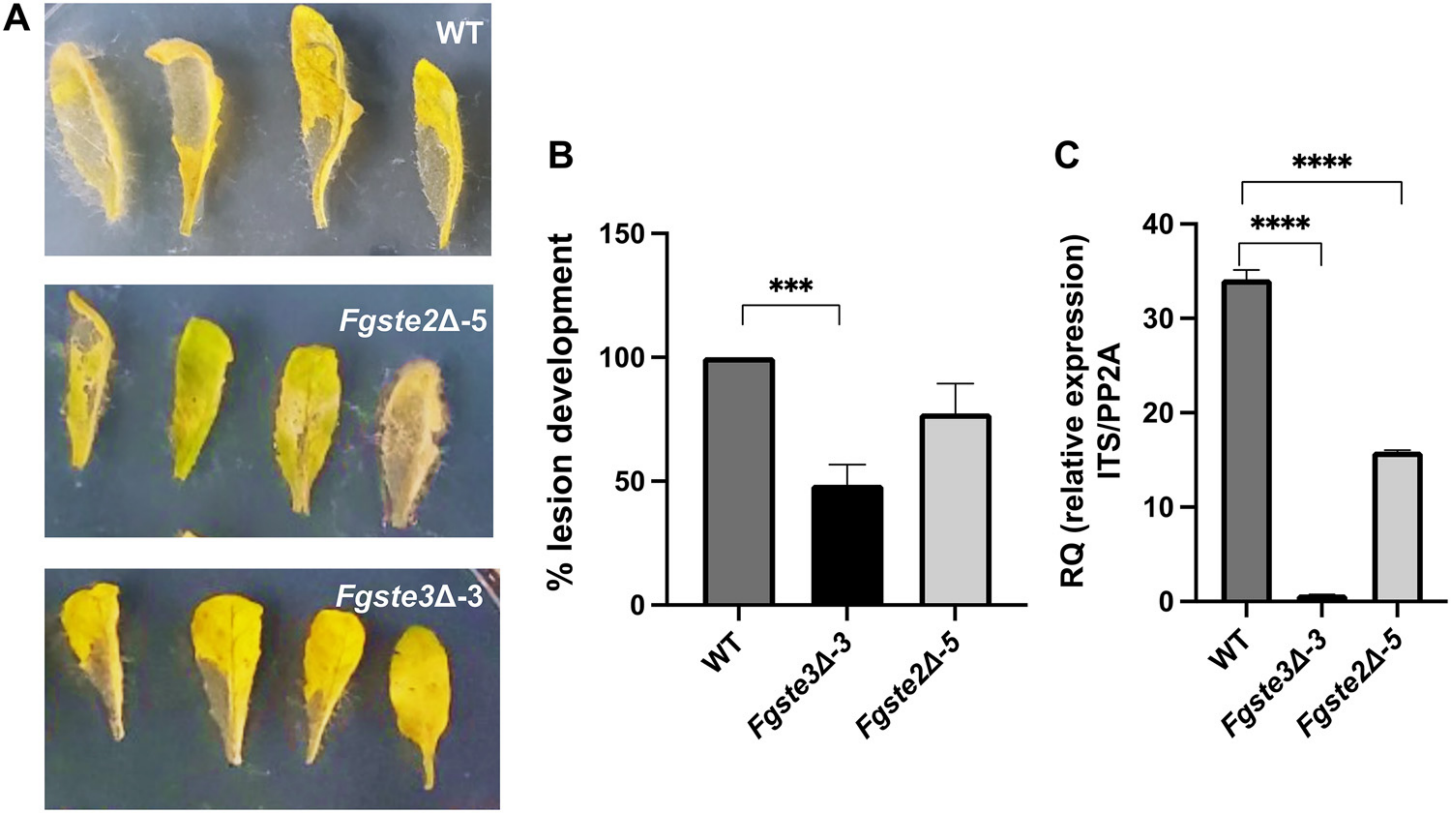
Deletion of *FgSTE3* leads to decreased F. graminearum pathogenicity against *A. thaliana*. (A) Representative images of *A. thaliana* leaf infected with F. graminearum strains. (B) Assessment of lesion development on *A. thaliana* leaves. Leaves were infected with the wild-type, *Fgste3Δ-3*, and *Fgste2Δ-5* strains and quantified 3 days postinfection using Image J software. (C) Assessment of F. graminearum infection via quantitative PCR. Quantitative PCR was performed with genomic DNA isolated from Arabidopsis leaves infected with the wild-type, *Fgste3Δ-3*, or *Fgste2Δ-5* strain. The relative expression (RQ) was measured with the Fusarium *ITS2*, with respect to the Arabidopsis *PP2A.* The experiment was performed three times (three biological replicates with *n* = 12 for each) with similar results. Error bars denote the standard deviation. The statistical analysis was performed using Student’s *t* test (****, *P* < 0.0005). Size bar = 1 cm.

To further investigate whether *FgS*te3 is involved in mediating fungal pathogenesis, a coleoptile infection assay was performed on the wild-type and *Fgste3Δ-3* strains. This assay has been shown to be an effective, reliable, and easily quantifiable method to investigate the pathogenicity and extent of Fusarium head blight infection (24, 31). The *Fgste3Δ-3* strain showed an average 50% decrease in lesion length compared to the wild-type strain (Fig. 3A and B). This trend is consistent with results arising from an *A. thaliana* leaf infection assay, in which decreased lesions for *Fgste3Δ-3* were observed, compared to the wild-type strain (Fig. 4A and B). A quantitative polymerase chain reaction (qPCR) analysis for fungal biomass quantification in the *A. thaliana* assay showed a 50% decrease in the absence of *Fg*Ste2 and a 99% reduction in the absence of *Fg*Ste3 (Fig. 4C). In the case of *Fg*Ste3 deletion, the fact that such a small amount of pathogen could elicit the observed lesion at all supports the claim that many of the lesions arise from host-mediated responses, leading to cell death.

Consistent with this, investigations of pathogenesis on flowering wheat heads yielded a decrease in percent infected spikelets for both the *Fgste3Δ* and *Fgste2Δ* deletion mutants (Fig. S4A), although these differences were not deemed statistically significant, compared to the wild-type under the conditions tested. The production of the mycotoxin DON, a Fusarium virulence factor and a critical component in fungal infections on wheat (32), was also assessed for the deletion mutants. Extracts were taken from suspension cultures of the wild-type, *Fgste3Δ*, and *Fgste2Δ* deletion strains grown on a medium that induces production of trichothecenes. Consistent with the wheat head infection results, both deletion mutants produced lower levels of DON, on average (Fig. S4B), with *Fgste3Δ-3* yielding a reduction of 30%, compared to the wild-type. The *P* value was slightly above the statistical significance cutoff (*P* = 0.069).

10.1128/msphere.00456-22.4 FIG S4 Wheat head infection assays and DON quantification. (A) Flowering wheat heads were point inoculated with the conidia of the wild-type and mutant strains *Fgste3Δ-3* and *Fgste2Δ-5.* The infected plants were transferred to a contained misting facility and monitored for the development of disease symptoms, such as spikelet discoloration. Data were collected when heads in the control treatment group exhibited approximately >90% infection. (B) DON quantification of the wild-type and *Fgste3Δ-3* and *Fgste2Δ-5* mutants grown in liquid culture with tricothecene-inducing media. Download FIG S4, TIF file, 1.3 MB. © Crown copyright 2022. 2022 Crown https://creativecommons.org/licenses/by/4.0/ This content is distributed under the terms of the Creative Commons Attribution 4.0 International license.

### Activation of the CWI-MAPK pathway by peroxidase is mediated by *Fg*Ste3 and *Fg*Ste2.

**FIG 5:**
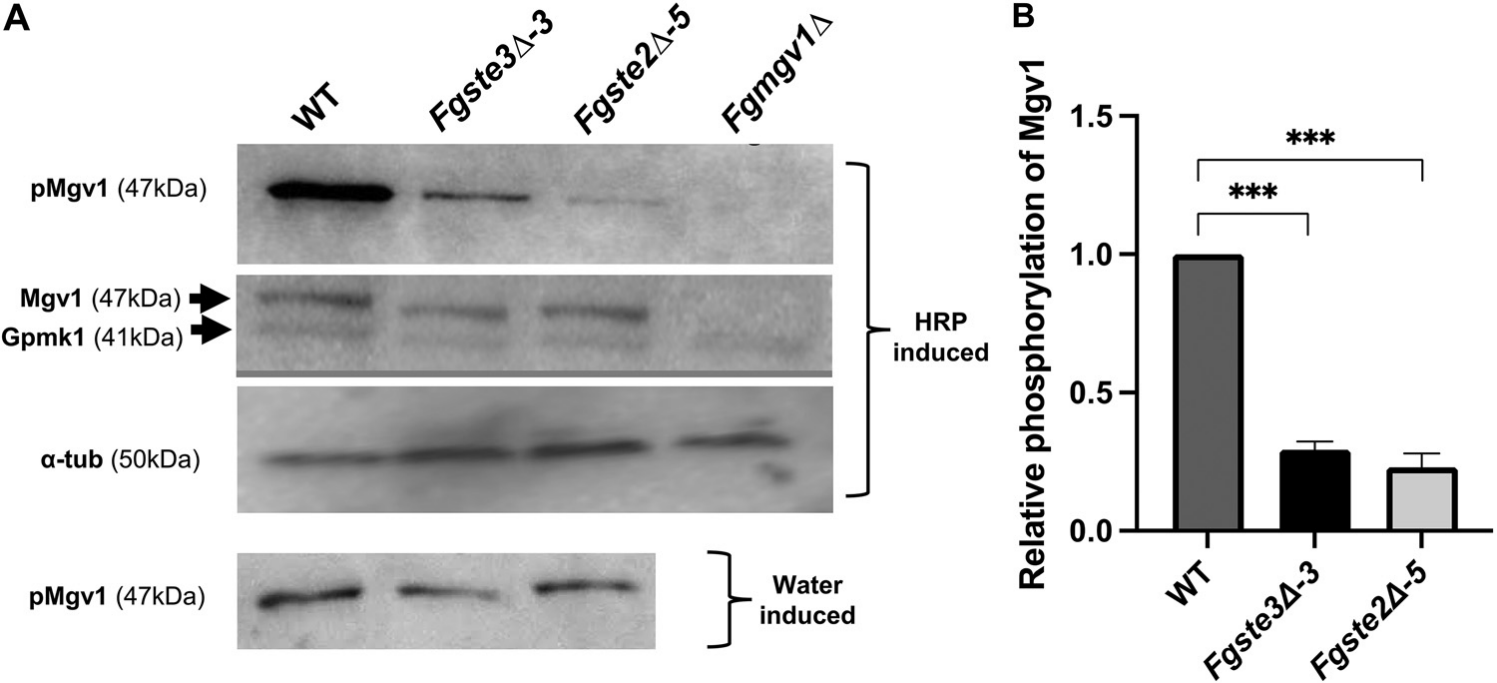
Activation of the CWI-MAPK pathway by peroxidase is mediated by *Fg*Ste3 and *Fg*Ste2. (A) Representative images of a quantitative Western blot to probe CWI pathway activation by tracking the phosphorylation of Mgv1 and total MAPK isolated from wild-type and mutant strains *Fgste3Δ-3*, *Fgste2Δ-5*, and *Fgmgv1Δ.* The conidia were grown for 48 h in regular PDB culture and were treated with commercially available HRP or a water control for 1 h before total protein extraction. For the normalization of the quantification, α-tubulin was probed. The molecular weights of the detected proteins are indicated on the blot. (B) The intensity of phospho-Mgv1 was quantified and normalized to tubulin, with relative intensities compared to the wild-type (***, *P < *0.0005). Quantification analysis was performed using ImageJ software. The data represent averages of three independent experiments. Error bars represent the standard deviation. The statistical analysis performed using Student’s *t* test.

It has previously been shown that the presence of peroxidase activates the CWI-MAPK pathway in F. graminearum (24). However, the roles of *Fg*Ste3 and *Fg*Ste2 in transducing this signal across the membrane to the CWI-MAPK have not been tested. Here, the phosphorylation of the CWI-MAPK pathway component Mgv1 was assayed using the phospho-p44/42 antibody in the presence and absence of *Fg*Ste3 or *Fg*Ste2. The phosphorylation of Mgv1 (expected molecular weight [MW] of 47 kDa) showed a 3-fold decrease in both mutants, compared to the wild-type (Fig. 5A and B; Fig. S5). Gpmk1 (expected MW of 41 kDa; MAP kinase in the filamentous signaling pathway that is also detectable by the phospho-p44/42 antibody [24]) was not detected using the phospho-p44/42 antibody. However, neither of the relative total amounts of Mgv1 or Gpmk1 varied from the wild-type levels in the knockout strains when probed with the total p44/42 antibody or with the water control. The *Fgmgv1Δ* strain served as a negative control and validated the detected 47 kDa band as Mgv1. These results indicate that the deletion of either *FgSTE3* or *FgSTE2* individually decreases the phosphorylation of Mgv1 to some extent, compared to the wild-type.

10.1128/msphere.00456-22.5 FIG S5 Probing of Mgv1 phosphorylation. Biological replicates of quantitative Western blot to probe CWI pathway activation by tracking the phosphorylation of Mgv1 isolated from the wild-type and mutant strains *Fgste3Δ-3*, *Fgste2Δ-5*, and *Fgmgv1Δ.* The conidia were grown for 48 h in regular PDB culture and were treated with commercially available HRP for 1 h before total protein extraction. In contrast, the band with water control showed no variation in the wild-type and mutants, as shown in Fig. 5A. Download FIG S5, TIF file, 1.5 MB. © Crown copyright 2022. 2022 Crown https://creativecommons.org/licenses/by/4.0/ This content is distributed under the terms of the Creative Commons Attribution 4.0 International license.

### Comparative transcriptomic analysis of the regulation of *F. graminearum* gene expression by peroxidase and *Fg*Ste3.

**FIG 6:**
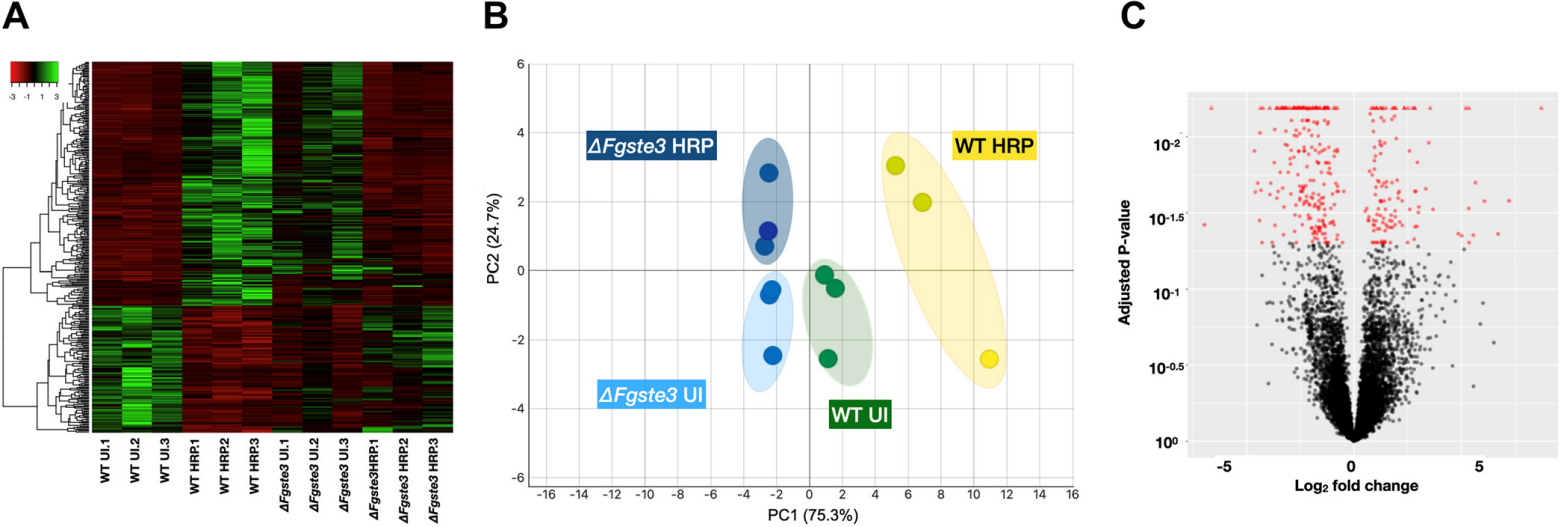
Transcriptomic overview of F. graminearum responses in the presence and absence of peroxidase and *Fg*Ste3. (A) Heat map showing the differential gene expression for different strains and conditions highlighting varying levels of upregulation and downregulation. (B) PCA plot showing the clustering of three biological replicates for each representative condition. (C) Volcano plot showing upregulated responses in the wild-type + HRP versus the wild-type uninduced pairwise comparison.

RNA-Seq reads were mapped to the genomic sequence of F. graminearum, resulting in 99.3% of the reads being successfully mapped. The different strains and treatments analyzed resulted in limited distinguishable changes to the transcriptome (Fig. 6A). After the normalization of the read counts, a total of 383 differentially expressed genes (DEGs) were identified for three pairwise comparisons relevant to this analysis, based on adjusted *P* values of ≤0.05 (Table 1; Data Set S1). The bulk (96.9%) of the DEGs arose from the comparison of the wild-type strain in the presence and absence of HRP. Consistent with this, the transcriptomic responses were most clearly divided based on the presence or absence of HRP and were found to be contributing 75.3% of the variance in a principal components analysis (PCA) (Fig. 6B).

**TABLE 1:** Total numbers of DEGs upregulated or downregulated arising from each pairwise comparison considered in this study

DEG regulation	Pairwise comparison		
	Wild-type + HRP/wild-type uninduced	*Fgste3Δ-3 *+ HRP/wild-type + HRP	*Fgste3Δ-3* uninduced/wild-type uninduced
Up	127	4	1
Down	243	4	4

10.1128/msphere.00456-22.7 DATA SET S1 Transcriptomic RNA-Seq data. Download Data Set S1, XLSX file, 0.4 MB. © Crown copyright 2022. 2022 Crown https://creativecommons.org/licenses/by/4.0/ This content is distributed under the terms of the Creative Commons Attribution 4.0 International license.

*Comparison 1: wild-type* F. graminearum *in the presence versus the absence of HRP*.

The pairwise comparison of the DEGs from the wild-type strain in the presence and absence of HRP was investigated to obtain broader insight into the genes potentially contributing to the HRP-induced chemotropic response in F. graminearum. While almost twice as many genes were downregulated as were upregulated (Table 1; Fig. 6A), a volcano plot analysis of the DEGs emphasizes the stronger nature of the upregulated responses (Fig. 6C). This is consistent with the idea of HRP inducing significantly increased expression of elements required to elicit chemotropism. Thus, the focus of this particular analysis is on the upregulated genes.

A gene ontology analysis of the upregulated genes highlighted the significant modulation of integral membrane transport activity (Table 2). F. graminearum is well-known to employ transporters to pump out the DON mycotoxin that it produces, depositing it on host cells (33). Major Facilitator Transporters are also known to act as energy centers that obtain nutrition that the pathogen needs for its survival (34). The best representative hits here include one of the top 10 most highly upregulated genes, the Major Facilitator Superfamily (MFS) transporter, *FGSG_13980*, although its function is not clearly defined (Table 2). Also notable is the significant upregulation of the MFS family pantothenate transporter Liz1 (*FGSG_04217*), which is known to contribute to septa formation and cellular development. Mutants of this gene have been shown to be defective in cell elongation and cell division in S. pombe (35).

**TABLE 2:** List of genes upregulated in the wild-type + HRP versus wild-type uninduced comparison

Gene identifier	Description	log_2_-fold change	*P* value
Membrane transporters			
*FGSG_13980*	The Major Facilitator Superfamily (MFS)	40.473	0.0008059030136
*FGSG_07502*	Transmembrane amino acid transporter	32.113	0.001594424155
*FGSG_05731*	Major Facilitator Superfamily transporter	11.643	2.43E−05
*FGSG_00195*	Monocarboxylate transporter 2	6.029	0.0008284057597
*FGSG_04217*	Pantothenate transporter liz1	5.969	0.0001984533517
*FGSG_04426*	Major Facilitator Superfamily transporter	2.853	0.0003898785871
*FGSG_04095*	Na(+)/H(+) antiporter 1	2.609	0.0004091487212
*FGSG_00924*	The Major Facilitator Superfamily	2.394	0.0006870875037
Cell wall remodeling			
*FGSG_03616*	6-hydroxy-d-nicotine oxidase	46.812	0.001572254889
*FGSG_03925*	Alpha/beta hydrolases	40.945	0.001858777969
*FGSG_13343*	NBD_sugar-kinase_HSP70_actin	7.06	1.70E−05
*FGSG_00659*	Endoplasmic reticulum mannosyl-oligosaccharide -alpha-mannosidase	2.446	0.001764702055
Pathogenesis			
*FGSG_01586*	Retinol dehydrogenase 14	4.152	0.0006120705837
*FGSG_11438*	Ankyrin repeat	3.148	0.0006318247356
*FGSG_04314*	ATP-dependent Clp protease ATP-binding subunit	2.829	0.0004512020458
*FGSG_07493*	Sensor gacS	2.328	0.0009534158274
*FGSG_05147*	Putative SCRAMM family adhesin clumping factor ClfB	2.111	0.001365630372
Peroxisomes			
*FGSG_07104*	Peroxisome biosynthesis	2.398	8.06E−05
*FGSG_00724*	Peroxisomal biogenesis factor 2	2.028	0.001092628833
*FGSG_05596*	Peroxisomal biogenesis factor 6	1.866	0.001059428996
*FGSG_01174*	Peroxisomal targeting signal receptor	1.677	7.47E−05
Mitochondria			
*FGSG_12693*	Altered inheritance of mitochondria 32	7.087	0.0005160525821
*FGSG_01639*	Enoyl-delta isomerase mitochondrial	2.685	0.0001528329539
*FGSG_05197*	Fmp40 found in mitochondrial proteome	2.611	9.19E−05
*FGSG_11231*	MOSC mitochondrial	2.073	2.44E−05

With relevance to chemotropism, a wider variety of genes and their homologues involved in cellular development and cell wall organization are highlighted (Table 2). The upregulation of hippurate and alpha/beta hydrolases (*FGSG_03925*), which are known to be involved in cell wall remodeling in fungi, was detected (36). In addition, alpha-mannosidase (*FGSG_00659*), which is required for the synthesis of mannose rich-N-glycans and is one of the major constituents of cell wall biosynthesis in rapidly dividing cells, was also upregulated (37). The upregulation of NBD sugar-kinase HSP70 actin (*FGSG_13343*), which regulates the formation of actin filaments in cells, was noted (38). Finally, and perhaps most clearly related to chemotropism at this time, was the observed upregulation of 6-hydroxy-d-nicotine oxidase (*FGSG_03616*), which is involved in the production of hydrogen peroxide. In F. oxysporum, hydrogen peroxide has been shown to be directly involved in mediating peroxidase-stimulated hyphal chemotropism, based on its biosynthesis by the nicotinamide adenine dinucleotide phosphate (NADPH) oxidase (NOX) gene (39).

Further investigation of the DEGs revealed additional hits with more direct relevance to infection mechanisms but less potential relevance to chemotropism. These include the upregulation of the gene *FGSG_05147*, which is related to the SCRAMM family adhesin clumping factor ClfB. It is used primarily by bacterial species such as Staphylococcus to promote the attachment and invasion of host cells. A protein that contains ankyrin repeats (*FGSG_11438*) is required for host-mediated nitric oxide production, and is known to promote virulence in F. graminearum was also upregulated (40). The upregulation of the gene *FGSG_04314* was also notable. It shares 36% identity with a homologue of Clp protease, an ATP-binding subunit adaptor protein that is known to be involved in the regulation of virulence genes in S. aureus, and 39% identity with a homologue of ClpX in S. cerevisiae (Mcx1p), which is known to act as a molecular chaperone that contributes to thermotolerance (41–43). Also upregulated was *FGSG_07493*, related to the putative gacS sensor, which is known to be involved in the control of the production of secondary metabolites and extracellular enzymes involved in pathogenicity in Pseudomonas. In addition, it mediates the production of acyl-homoserine lactones, which are responsible for quorum sensing in bacteria (44, 45). Though quorum sensing in pathogenic fungi has not been extensively studied, this raises a novel hypothesis that HRP may be involved in initiating the transcription of genes that enable fungal communication. *FGSG_07493* also shares 90% identity with S. cerevisiae nik-1, which is known to interact with Cdc28 and to regulate the progression of the cell cycle (46). Finally, retinol dehydrogenase (*FGSG_01586*), which is known to oxidise retinol to retinal (a precursor for carotenoid biosynthesis and abscisic acid [ABA] production), was also upregulated (47–49). The ABA phytohormone is well-known to act as a fungal effector and to accentuate fungal disease severity.

Four peroxisomal genes were identified as upregulated, including a peroxisomal targeting signal receptor (*FGSG_01174*), peroxisome biosynthesis factor 2 (*FGSG_00724*), a protein involved in peroxisome biosynthesis (*FGSG_07104*) and peroxisomal biogenesis factor 4 (*FGSG_05596*). Peroxisomes have been implicated as critical regulators of pathogenicity in Fusarium, harboring glutathione transferases that aid in the detoxification of host-derived proteins and in the maintenance of redox homeostasis. This highlights a possible mechanism for peroxide detoxification that is employed by Fusarium to deal with oxidative stress (50). Peroxisomes also control secondary metabolism involving DON biosynthesis, siderophore biosynthesis, and cell wall integrity in fungi (51).

A variety of mitochondrial genes were upregulated, including the mitochondrial amidoxime-reducing component 1 (MOSC1; *FGSG_11231*) and the altered inheritance of mitochondria 32 (AIM32; *FGSG_12693*), the latter being a 2Fe-2S protein that functions in redox quality control (52). Additionally, two mitochondrial carrier proteins: Fmp40 (*FGSG_05197*), which is found in the mitochondrial proteome, and enoyl delta isomerase (*FGSG_01639*) were both upregulated. Although it is hard to predict in which morphogenetic changes these mitochondrial proteins might be involved, they are generally related to oxidative stress responses, based on mitochondria being major centers for the generation of hydrogen peroxide and reactive oxygen species (ROS), which are later taken up by the peroxisome. However, mitochondrial proteins have also been shown to directly influence fungal pathogenicity through phenotypic changes that involve cell wall modification, polysaccharide capsule modulation, the evasion of the host immune response, metabolic flexibility by alternating between carbon source utilization, and controlling cAMP/PKA signaling (53, 54).

Finally, gene enrichment analyses in Blast2GO and KOBAS (Fig. S6) highlighted significantly upregulated genes (enrichment ratio of 0.27) that are involved in the biosynthesis of the branched chain amino acids (BCAA) valine, leucine, and isoleucine. BCAAs have been shown to play important roles during Fusarium pathogenesis, with the mutants of genes specifically involved in leucine biosynthesis, such as *Fg*LEU2, *Fg*ILV2, and *Fg*ILV6, being attenuated in infection potential, conidiation, and mycelial development (55, 56). Gene enrichment further revealed that biologically, the majority of DEGs are associated with membrane transport, transcription by RNA pol II, carbohydrate metabolic processes, proteolysis, and cellular amino acid metabolic processes. With respect to molecular function, most DEGs fall into the oxidoreductase category/class, followed by hydrolases and nucleotide-binding.

10.1128/msphere.00456-22.6 FIG S6 Gene enrichment analysis for the wild-type + HRP versus wild-type uninduced (upregulated genes only) using KOBAS. The differentially expressed genes were grouped into different categories based on a pathway enrichment tool. Each row represents an enriched function, and the length of the bar represents the enrichment ratio, which is calculated as the quotient of the input gene number divided by the background gene number. In the KOBAS algorithm, clusters are divided according to the values calculated for the enriched terms. The colors of the bars represent different clusters and show the top 6 with the highest enrichment ratios and *P* values. (KOBAS, http://kobas.cbi.pku.edu.cn). Download FIG S6, TIF file, 2.4 MB. © Crown copyright 2022. 2022 Crown https://creativecommons.org/licenses/by/4.0/ This content is distributed under the terms of the Creative Commons Attribution 4.0 International license.

*Comparison 2: Fgste3Δ-3 strain versus the wild-type strain*, *both treated with HRP*.

The pairwise comparison of the effect of HRP in the presence and absence of *FgSTE3* was assessed to gain insight into the role of *Fg*Ste3 in mediating transcriptomic changes in F. graminearum cells exposed to peroxidase, and, ultimately, the mechanism underlying *Fg*Ste3’s regulation of chemotropism. Interestingly, only 8 DEGs were deemed significant in this comparison, based on our cutoff criteria (Table 3).

**TABLE 3:** List of differentially expressed genes in the *Fgste3Δ* + HRP versus wild-type + HRP comparison

Gene identifier	Description	log_2_-fold change	*P* value
Downregulated			
*FGSG_12829*	Hypothetical protein	−4.828	6.09E−06
*FGSG_04590*	Averantin oxidoreductase	−2.094	1.60E−06
*FGSG_04596*	O-methyl transferase B	−1.877	3.13E−05
*FGSG_05039*	*Putative PTH 11*	*−0.959*	*0.1623589568*
Upregulated			
*FGSG_06536*	l-pipecolate oxidase	7.003	4.92E−06
*FGSG_09354*	N amino acid transport system protein	5.936	1.66E−05
*FGSG_09118*	Hypothetical protein	5.469	1.59E−05
*FGSG_09001*	Transcription factor	1.543	1.03E−05

Significantly downregulated genes included two genes related to the production of fungal toxins during the infection process, including a sterigmatocystin 8-O-methyltransferase (*FGSG_04596*) and a gene related to isotrichodermin C15 hydroxylase (*FGSG_04590*), a cytochrome P450 monooxygenase CYP65A1 which has been previously described to be a part of a C16 gene cluster that is involved in terpenoid biosynthesis. The expression of these two genes has been shown to increase dramatically 72 h postinoculation (hpi) on barley and wheat, and thus, they play essential roles in plant infection (57).

Also notable is the downregulation of *FGSG_05039*, which shares 79% amino acid identity to a PTH11-like GPCR from Hypocrea virens (*Trichoderma*). PTH11 (*MGG_05871*) from M. oryzae is known to be involved in appressorium formation, contributing to the generation of turgor pressure and the entry of fungal hyphae into the cells, which potentially implicates HRP-stimulated *Fg*Ste3 directly in pathogenesis (58). Although the log_2_-fold change was barely 1.0 for this hit, and although its adjusted *P* value was above the cutoff (Data Set S1), it shows a consistent decrease in all replicates. GPCRs are generally expressed at low levels, making their transcriptional detection challenging (59). For instance, even though *FgSTE3* was knocked out of the *Fgste3Δ-3* strain, because of its low constitutive expression in the wild-type strain, it did not fulfill the cutoff criteria that was set for this transcriptomic analysis and was not considered to be a downregulated gene, compared to the wild-type.

Of the upregulated genes, only l-pipecolate oxidase (*FGSG_06536*) was noted as an enzyme that was linked to lysine catabolism and protection against hydrogen peroxide stress (60). Thus, ultimately, while this pairwise comparison provides additional information regarding the potential regulatory roles of activated *FgSTE3* in infection, little insight was gained regarding the mechanism underlying its role in chemotropism.

*Comparison 3: Fgste3Δ-3 strain versus the wild-type strain*, *both untreated*.

Finally, the pairwise comparison of *Fgste3Δ-3* to the wild-type strain in the absence of any treatment was assessed to obtain further insight into the constitutive role of *Fg*Ste3. Constitutive activity refers to any basal level of signaling activity that a GPCR elicits in the absence of ligand stimulation. Many GPCRs are well-known to elicit constitutive activity (61). However, based on the cutoff criteria, only 5 genes were shown to be significantly differentially regulated upon the deletion of *Fg*Ste3 (Table 4), suggesting that *Fg*Ste3 has minimal, if any, constitutive role under these assay conditions.

**TABLE 4:** List of differentially expressed genes in *Fgste3Δ uninduced vs wild-type uninduced* comparison

Gene identifier	Description	log_2_-fold change	*P* value
Downregulated			
*FGSG_12651*	Conserved hypothetical protein	−4.068	5.77E−06
*FGSG_11101*	Hypothetical protein	−3.215	7.25E−06
*FGSG_08852*	Putative cryptochrome DASH	−2.474	6.23E−08
*FGSG_04576*	Conidial development transcriptional regulator fluffy	−1.033	3.35E−06
Upregulated			
*FGSG_01936*	Cutinase transcription factor 1 alpha	0.663	2.39E−05

Nonetheless, the one upregulated gene in the *Fgste3Δ-3* strain (albeit with a log_2_-fold change of only 0.6) was the cutinase transcription factor 1-α (*FGSG_01936*), which has been shown to be dispensable in F. oxysporum virulence (62). Thus, the relevance of the repression of this gene by constitutive *FgSTE3* remains enigmatic. With respect to the few downregulated hits detected, *FGSG_04576* is a homologue of the major regulator of conidiation in Neurospora crassa and is known as Fluffy. Fluffy is also known to directly activate a developmentally-regulated hydrophobin gene that is involved in osmotic stress tolerance (63). Finally, a gene related to the putative *Drosophila*, *Arabidopsis*, *Synechocystis*, human (DASH) cryptochrome (*FGSG_08852*), which is known to be involved in DNA photorepair and in the regulation of conidiation in the gray mold fungus Botrytis cinerea (64, 65), was also downregulated. Together, these results point toward a limited role for constitutive *Fg*Ste3 in stress sensing and in the regulation of conidiation and photoreception, although these findings remain to be validated functionally.

## DISCUSSION

F. graminearum is an aggressive pathogen of cereal crops that continues to adapt to the current line of antifungal agents (66). Prior studies emphasized dual roles for Ste2 and Ste3 in many of the biological processes that they regulate across fungal species (2–9, 22). The present study lends further evidence to this, demonstrating the involvement of the *Fg*Ste3 receptor in mediating chemotropism toward host-secreted peroxidases and virulence in its host, similar to activities previously documented for the opposite mating type receptors *Fg*Ste2, *Fo*Ste2, and *Vd*Ste2 (15, 23, 24).

The underlying mechanisms mediating the chemotropic responses for either *Fg*Ste2 or *Fg*Ste3 remain somewhat enigmatic. Previously, peroxidase induction of the CWI-MAPK pathway was demonstrated for F. graminearum, F. oxysporum, and V. dahliae (15, 23, 24). Here, this was extended by confirming the involvement of *Fg*Ste2 and *Fg*Ste3 in mediating this signal from peroxidase to Mgv1 in the CWI-MAPK pathway, with observed decreases in Mgv1 phosphorylation for both peroxidase-stimulated mutant strains (Fig. 5). With Mgv1 previously being shown to be important in fungal hyphal growth and pathogenicity (67, 68), these results are consistent with the observed reduction in chemotropic responses (Fig. 1) (24).

Further consideration of the chemotropic results emphasizes that the responsiveness of this homothallic F. graminearum fungus to pheromones is consistent with responses observed previously for the asexual F. oxysporum (22). This includes the observation that receptor deletion strains respond only to the opposite pheromone (Fig. 1). Furthermore, it is notable that each independent deletion is close to being fully effective in inhibiting peroxidase-directed chemotropism (Fig. 1). Because only one of the two pheromone receptors is deleted in each mutant strain, it follows that the remaining receptor is incapable of mediating the peroxidase-stimulated chemotropic response, even a partial one, on its own. That is to say that neither of the two receptors can compensate for the loss of the other receptor. This raises the novel hypothesis that *Fg*Ste2 and *Fg*Ste3 work together to mediate the chemotropic response to peroxidase. Speculatively, this interdependence may be the result of independent signaling events elicited by each individual receptor. In this case, one might expect that changes to the chemotropic responses would be cumulative, rather than absolute, with single receptor deletion. Additionally, in that both receptors stimulate the CWI pathway, the interdependence could be consistent with a requirement for the formation of a peroxidase-stimulated heterodimer complex between *Fg*Ste2 and *Fg*Ste3 in the cell membrane. GPCR dimers (hetero and homo), as well as higher order oligomers, are well-documented to play important roles in modifying GPCR signal transduction (69), including a prior report of Ste2p homooligomerization in yeast mating (70).

Host infection assays were used to assess the extent of fungal pathogenicity related to the *Fg*Ste3 receptor. Coleoptile infection data showed a consistent and significant decrease in lesion development, yielding a reduction of almost 50% compared to the wild-type (Fig. 3), similar to observations made previously for the *Fgste2Δ* strain (24). In contrast, slightly decreased values for the average infections of wheat heads and lower accumulations of DON for both *Fgste3Δ-3* and *Fgste2Δ-5* were not found to be statistically significant (Fig. S4). While confirming the contribution of *Fg*Ste3 to virulence, the comparison of results from wheat heads and wheat coleoptiles suggests that different routes of pathogenesis may be more or less reliant on *Fg*Ste2 and *Fg*Ste3 (i.e., potentially more prominent in stalk infections than in wheat head infections) (24). Additionally, in plants, salicyclic acid (SA) defense is generally predominant against biotrophic pathogens, and jasmonic acid and ethylene (JA/ET) allow resistance to necrotrophic pathogens. Both the SA and JA/ET defense responses can also attenuate each other or work in a synergistic way (71, 72). Thus, with F. graminearum being a hemi-biotroph, plants could elicit a wide range of variable defense responses that potentially differ in wheat head and coleoptile tissues. Alternatively, the difference between wheat head and coleoptile infection results may simply be a product of the assay systems. With the conidia being deposited directly at the site of infection on the kernel of a wheat head with a pipettor, any significant need for chemotropism may be eliminated. In contrast, the coleoptile assay sees the conidia presented to the leaf indirectly on cotton fibers dipped in conidia suspensions and then wrapped loosely around the wound sites. As such, chemotropism could be necessary for the hyphae to even make initial contact with the coleoptile leaf.

The results loosely linking DON accumulation to the pheromone receptors opens the question of the broader impact and role of *Fg*Ste3 in F. graminearum infection. To glean further insight into the mechanisms related to peroxidase-induced, *Fg*Ste3-mediated chemotropism and virulence, a comparative transcriptomic study was completed. At the time of writing, this is the first global transcriptomic RNAseq study carried out in the context of the peroxidase-mediated chemotropism of Fusarium, to our knowledge. Broadly speaking, the DEG analysis highlighted a somewhat limited but fairly diverse array of modifications arising from treatment with peroxidase. Detailed DEG interpretations are largely included above in the Results section. However, more generally, with respect to oxidation and as a known component of the plant pathogen defense response (24, 49), it is not surprising that F. graminearum would have multiple varied responses to treatment with peroxidase. For example, transcription factors related to purine utilization and the cutinase enzyme used for plant cutin degradation were seen to be upregulated in the wild-type strain in response to peroxidase. As well, ornithine cyclodeaminase, which favors the conversion of ornithine to proline during the stress response was also upregulated. Notably, ornithine is an essential component in the synthesis of DON (73, 74).

With respect to chemotropism, HRP induced a selection of genes involved in cell wall remodeling and with relevance to hyphal redirection during growth. Many additional changes linked to pathogenic transitions that contribute to the switch from the benign phase to the beginning of the pathogenic phase, both for penetration and for obtaining nutrition, were also observed. Previous transcriptomic studies of Fusarium interactions with host plants have also highlighted the upregulation of peroxidases, which in turn activate other ROS-related genes. The expression of various pathogen associated molecular patterns (PAMPs) is also known to induce ROS bursts in host plants (75). In yeast, ROS stress is known to activate various signaling pathways and Rho GTPases, the guardians of the cell wall integrity pathway, with Rho1 controlling cell polarity (76).

In contrast, only a limited number of transcriptional changes were detected upon *Fg*Ste3 deletion. In the presence of HRP, genes involved in mycotoxin biosynthesis and appressorium formation were downregulated in the absence of *Fg*Ste3, linking important virulence-related events to this receptor. The fact that this transcriptomic analysis did not reveal a connection to chemotropic-related machinery, such as CWI pathway components or cell wall modifying enzymes, may be due to underlying mechanisms acting at the post-transcriptional level, generally low expression levels of signal transduction machinery, or strong general HRP effects masking the weaker Ste3-related mechanisms. Alternatively, the 24 h post HRP-induction time point selected for this transcriptomic analysis may not have been optimal for the detection of *Fg*Ste3-related events.

Overall, this report demonstrates a role for *Fg*Ste3 in chemotropism and virulence. This is consistent with previous reports that highlight similar roles for Ste2 (15, 23, 24). While the transcriptomic analysis shed only limited light on the mechanisms by which *Fg*Ste3 might be mediating chemotropism, a clear relationship between HRP stimulation and cell-wall remodeling is highlighted. As well, potential roles for *Fg*Ste3 in mycotoxin production and appressorium formation are noted. Finally, evidence that *Fg*Ste2 and *Fg*Ste3 are interdependent on each other for the perception of the peroxidase-derived signal and the elicitation of the chemotropic response is highlighted. Experiments investigating the interdependence of the receptors’ roles in chemotropism, along with the elucidation of the nature of the peroxidase-derived ligand stimulating the receptors, are ongoing in our labs. Together, this research will enhance our understanding of how two related GPCRs can play so many different roles across mating, host perception, and virulence.

## MATERIALS AND METHODS

### Chemicals.

All chemicals were obtained from Sigma-Aldrich (Burlington, MA, USA) unless otherwise indicated below.

### Fungal strains, preparation of conidia and protoplast production.

F. graminearum wild-type strain DAOM 233423 (also known as GZ 3639) was provided by C. Babcock of the Canadian Collection of Fungal Cultures (CCFC/DAOM), Agriculture and Agri-Food Canada, Ottawa. The *Fgste2Δ-5* deletion strain arose from wild-type GZ 3639, as did *Fgmgv1Δ*, and both are described in detail in our prior work (24). The wild-type strain was inoculated into 200 mL of liquid carboxymethylcellulose (CMC) media from a frozen glycerol stock and was grown in a rotating shaker at 180 rpm for 4 days at 28°C in the dark. Macroconidia were harvested by filtering the culture through a double layer of sterile cheesecloth, and this was followed by centrifugation for 10 min at 1,500 × *g* at 4°C. The supernatant was discarded, and the conidia were washed twice with sterile water and then resuspended to obtain a concentration of approximately 7 × 10^8^ conidia per mL, as measured by a haemocytometer. For protoplast production, 1 mL of the conidia solution was inoculated into 100 mL of yeast peptone dextrose (YPD, BD Difco) media and grown for 8.5 h at 28°C with shaking at 170 rpm. When the germ tubes were observed to be approximately 1.5 times the length of the conidium, the mycelia were harvested by filtration through double layered Mira cloth and were washed with 50 mL of sterile water and 30 mL of 1.2 M KCl. The mycelia were transferred to 20 mL of protoplasting solution (500 mg Driselase [Sigma, D-9515], 200 mg Lysing enzyme [Sigma, L- 1412], and 200 mg Yatalase [TaKaRa Biotech, T017] dissolved in 20 mL of 1.2M KCl). This solution was incubated at 28°C with shaking at 170 rpm and was monitored every 10 min for progression. At 40 min, the protoplasts were separated from the mycelial debris by overlaying the protoplast mixture with trapping buffer (0.6 M Sorbitol, 100 mM Tris-HCl, pH 7.5), followed by centrifugation at 1,500 × *g* for 10 min at 4°C. The protoplast layer was transferred to a new tub and washed three times with 30 mL of 1.2 M KCl with centrifugation at 1,500 × *g* for 10 min at 4°C between washes. The protoplasts were resuspended in 5 mL of 1× STC buffer (1.2M Sorbitol, 10 mM Tris-HCl [pH 8.0], 50 mM CaCl_2_), yielding a final concentration of 10^7^ to 10^8^ per mL. Dimethyl sulfoxide (DMSO, 7% final concentration) was added, and aliquots (200 μL) were stored in 1.5 mL tubes at −80°C.

### Crispr/Cas9 knockout of *STE3* in F. graminearum.

The entire coding sequence of *STE3* (NCBI GenBank accession: FGSG_07270) was deleted as described previously (77). Briefly, a dual Cas9-gRNA (guide RNA) was used, where two separate crRNAs (CRISPR RNAs), C1 and C2, were designed to target selected protospacer sequences in the 5′ and the 3′ UTRs of (untranslated regions) *STE3*, respectively (Fig. S1; Table 5). The chop chop tool (https://chopchop.cbu.uib.no) was used to identify the best protospacer adjacent motif (PAM) sites that were recognized by the NGG sequence and were upstream and downstream of the *STE3* start and stop codons, respectively. A BLAST search for the selected protospacer sequences within the F. graminearum genome on the ENSEMBL Fungi database (https://fungi.ensembl.org/) was conducted to ensure that they displayed less than 15 base pairs (bp) of identity to any off-target locus in the genome. A selectable marker, hygromycin B (Hyg), was used to replace the STE3 gene in the Cas9 mediated gene deletion. A 1,500 bp segment, spanning a region of the pTrpC promoter and the hygromycin B phosphotransferase (hph) and referred to as the hygromycin repair template, was PCR amplified from the Prf-HU2 vector (78), using a designed primer set, namely, P1 and P2 (Table 1) and Phusion Hotstart polymerase (NEB, M0530S). The resulting PCR fragment was purified using a PCR purification kit (Qiagen, 28104), yielding the complete repair template, which was composed of a Hyg cassette flanked by 50 bp microhomology regions targeting coding regions for the *STE3* gene. The *in vitro* assembly of commercially available, Alt-R-CRISPR-Cas9 components from Integrated DNA Technologies (IDT) was carried out with Cas9 ribonucleoproteins (RNPs), the crRNAs, and tracrRNA (trans-activating CRISPR RNA). The F. graminearum strain DAOM 233423 protoplasts were thawed on ice, and 200 μL were transferred to a sterile 15 mL tube that contained the reaction mixture of the dual RNPs targeting the 5′ and 3′ end of the STE3 gene. Approximately 9 μg of the purified hygromycin repair template (150 μL in volume) and 25 μL of polyethylene glycol (PEG)-CaCl_2_ buffer (60% wt/vol PEG 4000, 50 mM CaCl_2_·2H_2_O, 450 mM Tris-HCl, pH 7.5) were added, and the mixture was incubated on ice for 50 min. Subsequently, 1.25 mL of PEG-CaCl_2_ buffer were added, and the mixture was incubated at room temperature for another 20 min. The mixture was diluted to a total volume of 4 mL via the addition of TB3 molten media (3 g/L yeast extract, 3 g/L Casamino acids, 200 g sucrose/L) and was incubated for 18 h at 25°C at 150 rpm to allow for the regeneration of the fungal cell walls. Finally, 300 μL of the transformed protoplasts were mixed with 15 mL of low melting point agar (Thermo Fisher, 16520050) supplemented with 100 μg/mL hygromycin and spread onto culturing plates. The plates were incubated at 28°C for 4 days, until mycelium emerged from the surface. Putative transformants were picked and subjected to two further rounds of selection on potato dextrose agar (PDA) plates supplemented with 150 μg/mL of hygromycin. Individual colonies were picked, and conidia were obtained by growing in CMC media. The conidia were harvested and stored at −80°C.

**TABLE 5:** List of primers and crRNA sequences

Name	Description	Sequence
P1^*a*^	5′ Hygromycin repair template	ATATATACTCAACCACTCACTCAAGAGCCTCAAAAAGCCTCTTCCACATC**TCGACAGAAGATGATATTG**
P2^*a*^	3′ Hygromycin repair template	AAAAAGGCACAAAAATCAAATAGGGTATCGCACGATGTACTTTTTGG**CCACTATTCCTTTGCCCTCGGACGA**
P3	Ste3 gene ORF-F	ATGGCCGATTCAATTCACTTG
P4	Ste3 gene ORF-R	CTAGCGTCGATATGTTTCCTC
P5	(Fusarium) ITS2 F	GTCGAGCTTCCATAGCGTAGTA
P6	(Fusarium) ITS2 R	CTACCTGATCCGAGGTCAACAT
P7	Arabidopsis PP2a (At 1G69960) F	AGTTCCAGAATCCAAACCAAC
P8	Arabidopsis PP2a (At 1G69960) R	CCTAGAGGCAACACAAACATC
C1	5’crRNA	AAAAAGCCTCTTCCACATCA
C2	3’crRNA	ATTCATCATCTATTCCGGGG

a The region in bold denotes hygromycin overlapping sequences.

### Fungal genomic DNA isolation and whole-genome sequencing.

Fungal conidia stocks of the transformants at −80°C were thawed on ice and grown in potato dextrose broth (PDB) media. Mycelia were collected from 2-day-old F. graminearum liquid cultures by filtration and ground into a fine powder in liquid nitrogen. Genomic DNA (gDNA) was then isolated from the ground tissue using an E.Z.N.A. Fungal DNA Mini Kit (Omega Biotek, D3390-01) and eluted in sterile water. The deletion of the target *STE3* sequence was confirmed by PCR, using primer set P1 and P2 for the hygromycin gene and P3 and P4 to show the absence of *FgSTE3* (Table 5). *FgSTE3* deletion was validated via whole-genome sequencing performed on a NovaSeq 6000 PE100 (5M reads) platform, following Illumina shotgun library preparation. The FASTQ files were uploaded and the data were analyzed using the Qiagen CLC-Genomics platform (v. 21.0.5), using the default settings. In brief, reads with an error probability of <0.05 (corresponding to a Phred score of ~15) and with more than 2 ambiguous bases were trimmed. Terminal nucleotides were trimmed by 10 bases from the 5′ end to limit sequencing bias, and IDT dual-index sequencing adaptors were removed, resulting in an average read length of 90.8 bases. The trimmed sequences were aligned to the reference genome (GCA_023242275.1) using the “Map Reads to Reference” tool in the CLC-Genomics Workbench with the default settings, resulting in a 90.5% mapping efficiency to the reference and an average coverage of 308.95-fold. In total, 3,366 positions in the reference genome (0.009%) were covered by no sequencing reads, including the 1,790 bp corresponding to the region of *FGSG_07270* (i.e., *FgSTE3*).

### Quantitative chemotropism plate assays.

Chemotropism plate assays were set up as described previously with minor modifications (24). Chemoattractant solutions (50 μL) that included either 4 μM horseradish peroxidase (HRP; Sigma, P8375) in water or 378 μM chemically synthesized (Bio Basics) F. graminearum a-factor (QKPGYPLSCTVM) or α-factor pheromones (WCTWKGQPCW) dissolved in 50% methanol (vol/vol) were loaded in plate wells against solvent controls (water or 50% methanol [vol/vol], respectively). The plates were incubated at 28°C in the dark for 14 h. The directional growth of conidial germ tubes (hyphal tips) was quantified with a stereomicroscope (ZEISS Axiocam ERc 5s). The chemotropic index calculations were obtained using the formula [*N*_test_ − *N*_solv_]/(*N*_total_) × 100, where *N*_test_ is the number of hyphae growing toward the chemoattractant solutions, *N*_solv_ is the number of hyphae growing toward the solvent control, and *N*_total_ is the total number of hyphae counted. For each interaction, a minimum of 500 hyphal tips were scored. The plotted data are the averages of 3 independent biological replicates (*n* = 500 hyphae/interaction/replicate; **, *P* < 0.001). The statistical analysis was conducted using Student’s *t* test.

### Wheat coleoptile infection assay.

The infection of germinating coleoptiles with the wild-type and *Fgste3Δ3* strains was carried out as previously described (24). Briefly, 16 Triticum aestivum cultivar “Roblin” seeds per F. graminearum strain to be tested were placed on 1/2 MS media in 0.7% (wt/vol) agar in water in autoclaved Magenta boxes and were stratified overnight at 4°C in the dark. The Magenta boxes were then placed in a growth chamber (Enconair chamber AC 60) with growth light and temperature conditions set to 20°C day and 16°C night, with a 16 h photoperiod (750 μmol photons/m^2^·s), and coleoptiles were grown to a height of 1 cm, with 12 to 16 seeds germinated per strain. Sterile scissors were used to cut 1 mm off the top of the coleoptile, and cotton thread soaked in a macro conidial suspension (2 × 10^5^ conidia per mL) was wrapped around the wound site. The Magenta boxes were then placed back into the growth chamber to allow for symptom development. After 10 days, the lengths of the infected lesions observed on the coleoptile stalks were measured for each strain. The averages of two independent biological replicate experiments are shown (compared to the wild-type strain; *n* = 18 for each experiment). The statistical analysis was conducted using a one-way analysis of variance (ANOVA).

### *A. thaliana* detached leaf assay with fungal biomass quantification.

Conidia concentrations for the wild-type and knockout strains were adjusted to 1 × 10^4^ conidia/mL. Briefly, 10 detached leaves from 3-week old *A. thaliana* Col-0 plants were transferred onto a petri-plate containing 1% agar. Wound inoculation on detached leaves of *A. thaliana* was conducted as previously described (79, 80). Plates were incubated in the dark at 28°C to simulate the conditions required for infection. The progression of disease and lesion development on leaves were monitored for 3 days. Fungal DNA isolation from *A. thaliana* leaves at 2-days postinfection was performed using a Phytopure plant DNA extraction kit, according to the manufacturer’s instructions (Amersham Biosciences, QC, Canada). DNA (30 ng) was applied to qPCR using PowerUP Syber Green Master Mix (Applied Biosystems). Primers of the fungal rDNA-ITS region (P5 and P6) and *A. thaliana* PP2A (AT1G69960; P7 and P8) were used for normalization to calculate the relative expression levels (Table 1). Two biological replicate experiments were averaged (*n* = 12 each) for the leaf assay, and 3 biological replicates were averaged for the qPCR assay.

### Wheat head infection assay.

A pathology test was performed by point inoculation on a susceptible variety of wheat (*cv.* Roblin) (81). Two independent biological repetition experiments were performed with 11 to 15 wheat spikelets per experiment.

### DON quantification.

Deoxynivalenol production was measured *in vitro* using a modified two-stage media protocol (81). Briefly, 5,000 conidia/mL were inoculated into 4 mL of 95% putrescine second-stage media (6.2 mM Putrescine di-hydrochloride, 22 mM KH_2_PO_4_, 0.8 mM MgSO_4_·7H_2_O, 85.6 mM NaCl, 116.8 mM sucrose, 108.6 mM glycerol [vol/vol], pH 4.0), and 5% glucose yeast-extract peptone first-stage media (GYEP; 56 mM NH_4_Cl, 8.1 mM MgSO_4_ 7H_2_O, 0.23 mM FeSO_4_·7H_2_O, 14.7 mM KH_2_PO_4_, 2 g/L peptone, 2 g/L yeast extract, 2 g/L malt extract, and 111 mM glucose) per well, with each containing one sterile nylon filter (Millipore #NY1H). Culture plates were incubated at 28°C and 160 rpm in the dark for 72 h. Mycelia were vacuum-dried and weighed. Culture supernatants were filtered (0.2 μM) and diluted to a final concentration of 15% MeOH. Trichothecenes were analyzed on a Shimadzu Prominence LC-20AD (Mandel) with a 100 μL injection on a Shimadzu SIL-20A HT Prominence autosampler. Samples were run using a 22.5% isocratic methanol:water mobile phase at a rate of 1 mL/min for 20 min on a Restek Pinnacle DB C18 column (5uM, 150 × 4.6 mm, Cat. number 9414565). Trichothecenes (DON) were monitored by UV at 220 nm and were reported as micrograms of toxin per milligram of mycelial tissue.

### Western blotting of MAPKs.

Approximately 10^5^ F. graminearum conidia for each respective strain were grown in PDB for 24 h at 28°C in the dark. HRP (0.05 μM) or an equivalent water control was then added to the growing culture and shaken for 1 h. The cells were lysed as previously described (82). The total protein (20 μg) of each sample was loaded and resolved on a 12% SDS polyacrylamide gel and transferred to a polyvinylidene difluoride membrane (PVDF, Bio-Rad) via wet electroblotting at 100 V for 2 h. The membranes were blocked for 1 h in 5% (wt/vol) nonfat, dried, skim milk in TBST buffer (50 mM Tris [pH 7.5], 150 mM NaCl, 0.05% [vol/vol] Tween 20) at 4°C. The membranes were subsequently incubated with either anti-p44/42 MAP kinase (1:1,000 dilution, M5670, Millipore Sigma) or anti-phospho p44/42 MAP kinase (1:1,000 dilution, Cell Signaling Technology, 9101) primary antibodies. Following a wash step, the membranes were incubated with HRP linked IgG secondary antibody for 1 h at room temperature for chemiluminescent detection (1:5,000 dilution, Cell Signal Technology, 7074S). For visualization, enhanced chemiluminescent substrate (Thermo Scientific, 32209) was added to the membranes, and the emitted light was captured using a GelDoc Imager and the Image Lab software package (Bio-Rad). The membrane was stripped and then re-probed for α-tubulin (1:1000, Santa Cruz Biotechnology, sc53030) as a loading control. Quantification was performed using ImageJ (83) (https://imagej.nih.gov/ij/, 1997 to 2018). Three independent biological repetitions of the experiment were averaged and analyzed using Student’s *t* test.

### Total RNA extraction and differential RNA-Seq analysis.

Approximately 2 × 10^5^ conidia of both the wild-type and the *Fgste3Δ-3* strains were grown in 2 × 20 mL of PDB for 3 days at 28°C in the dark. At this time, one culture of each strain type was induced with 0.05 μM HRP, whereas the other was left to grow uninduced. After a further 24 h, the mycelia from all of the cultures were harvested. For RNA extraction, 1 g of dried mycelial mass was frozen in liquid nitrogen and homogenized in 1 mL of TRIzol reagent, prepared according to the manufacturer’s instructions (Thermo Fisher Scientific). The InviTrap Spin Universal RNA Mini Kit (Stratec Molecular, Germany) was used to purify the total RNA from the TRIzol aqueous phase, according to the manufacturer’s protocol. The RNA concentration and purity were subsequently determined using a Nanodrop spectrophotometer ND-1000 (Thermo Scientific). The RNA-Seq libraries were prepared using a TruSeq Stranded RNALT Kit and were sequenced on an Illumina HiSeq 2500 platform, according to the manufacturer’s guidelines (Illumina, USA). The F. graminearum RNA-Seq data were analyzed using CLC Genomics Workbench, version 11.0.1 (Qiagen Corp.). The raw data were trimmed using Trimmomatic v0.39 (http://www.usadellab.org/cms/?page=trimmomatic), based on the default quality scores that were determined by the base caller error probability level (*P < *0.01). To estimate the expression levels, high quality RNA sequences were aligned to the F. graminearum RR1.36 genome that was annotated with genes and transcripts using Salmon v1.2.1 (https://combine-lab.github.io/salmon/) (84). A differential expression analysis was performed using SARTools v1.6.4 with the DESeq2 option and the parameters provided within the default template (85). The differentially expressed transcripts over the threshold false discovery rate (FDR) (86) of corrected adjusted *P* values of ≤0.05 were considered. Gene annotation and a GO enrichment analysis for F. graminearum were accomplished within the FungiDB database (https://fungidb.org/fungidb/) (87, 88) and the KOBAS database (http://kobas.cbi.pku.edu.cn) (89). The full RNA-Seq data set is available from the NCBI (Bioproject ID: PRJNA872394). Three biological replicates were used per condition.
