# Supplementary figures and images for: Fusarium graminearum Ste3 G-Protein Coupled Receptor: A Mediator of Hyphal Chemotropism and Pathogenesis
Source: mSphere. 2022 Nov 15;7(6):e00456-22. doi: 10.1128/msphere.00456-22 (PMC9769807; doi:10.1128/msphere.00456-22)

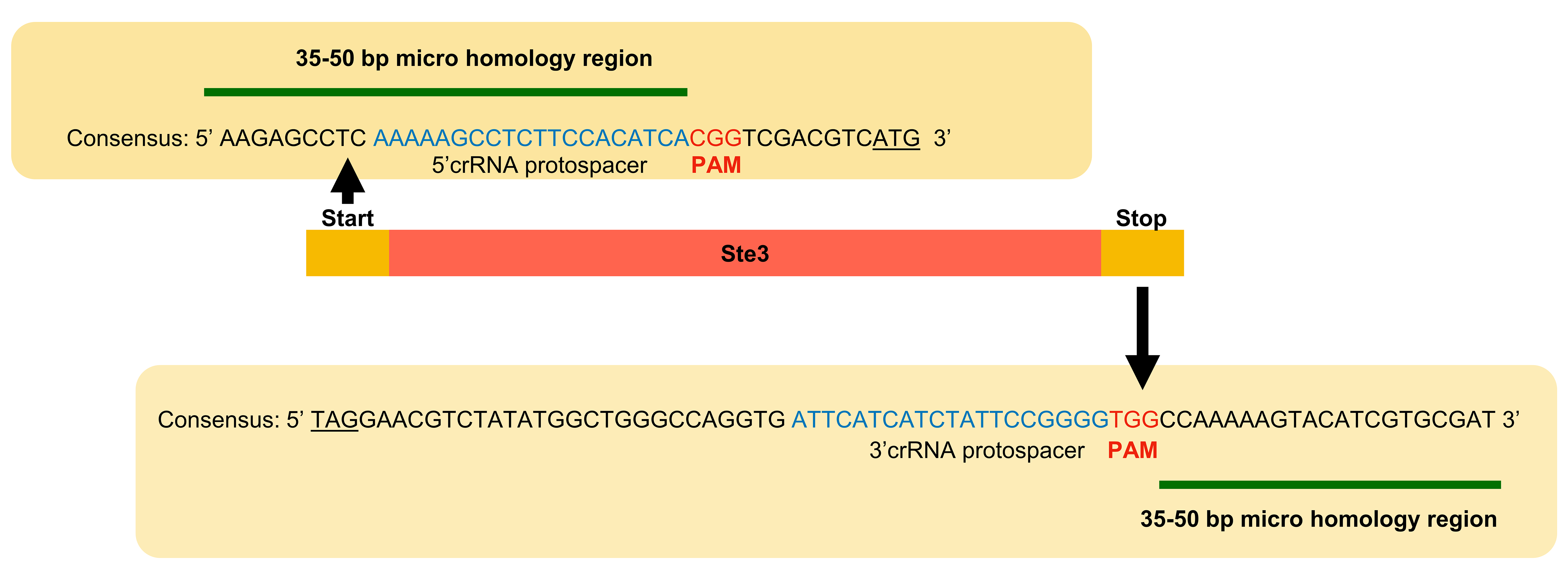

Supplement: FIG S1 [file msphere.00456-22-s0001.tif]

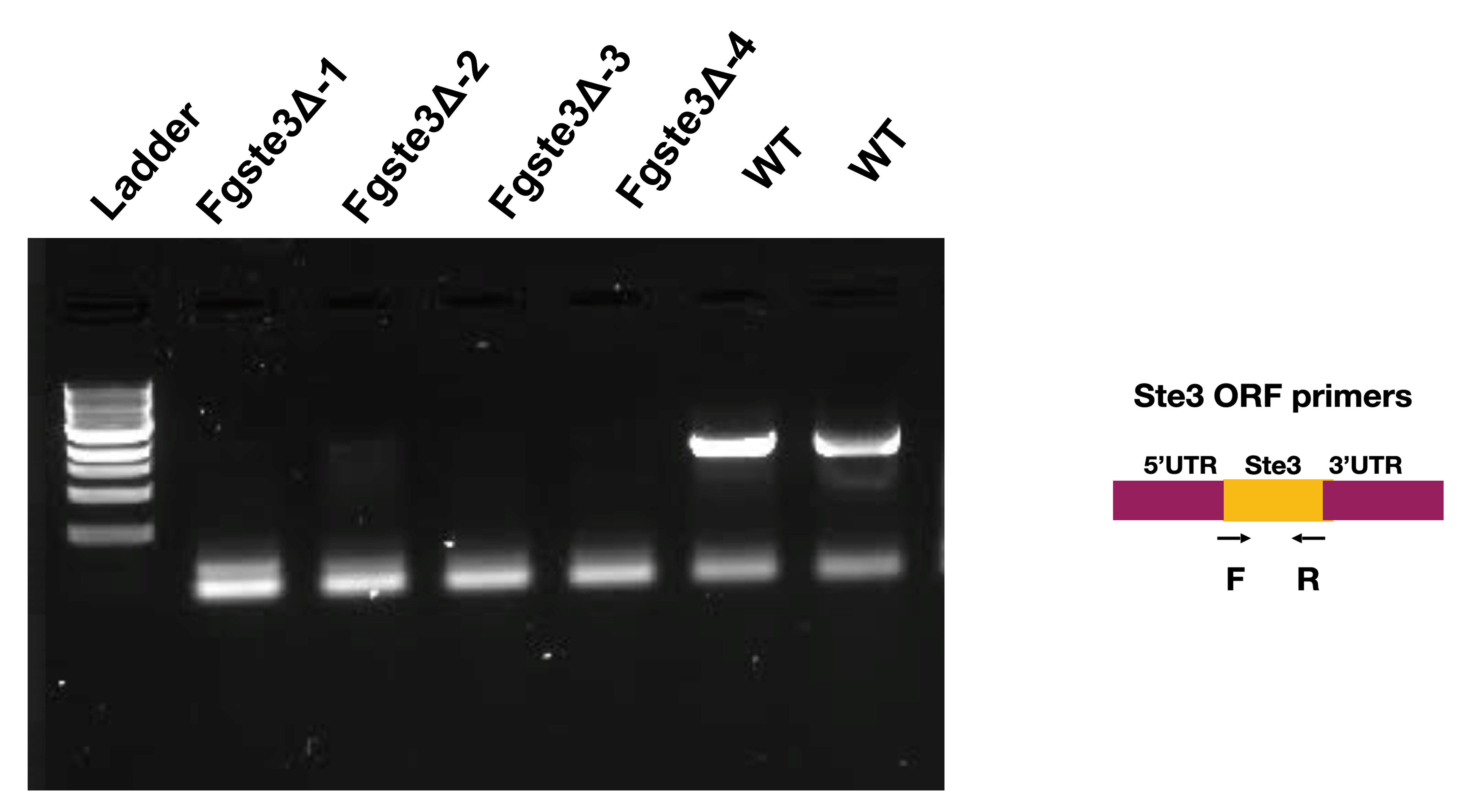

Supplement: FIG S2 [file msphere.00456-22-s0002.tif]

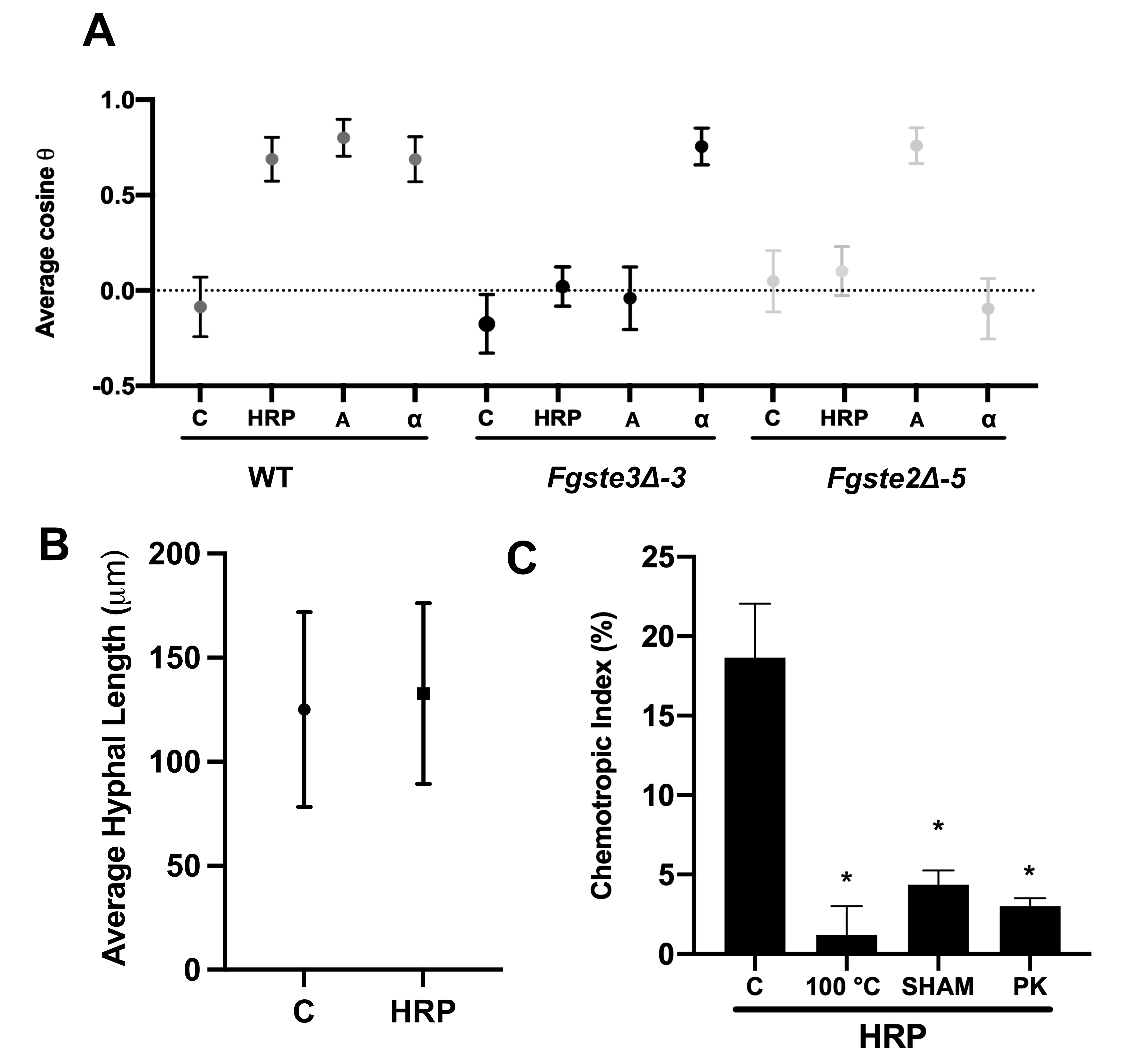

Supplement: FIG S3 [file msphere.00456-22-s0003.tif]

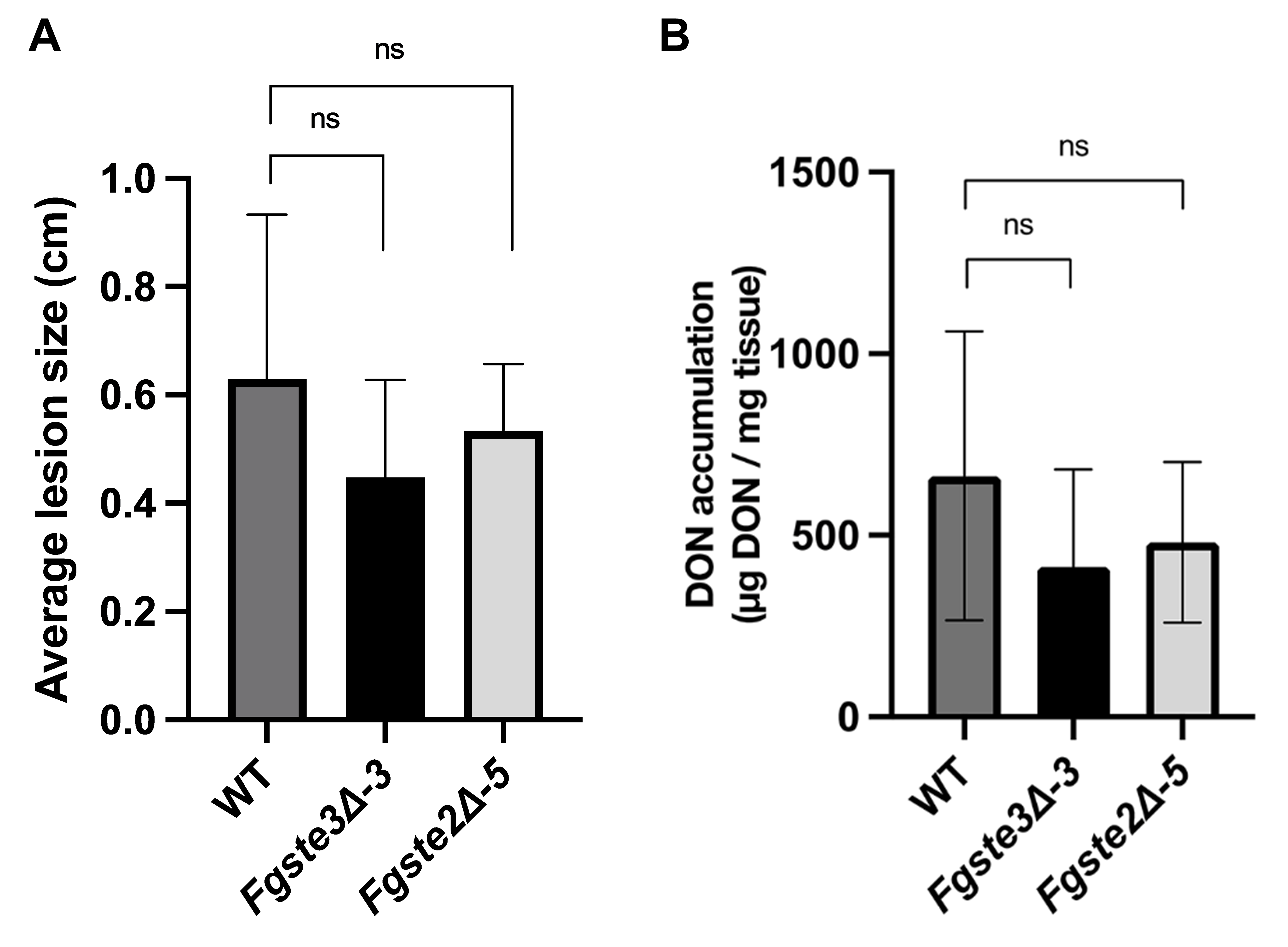

Supplement: FIG S4 [file msphere.00456-22-s0004.tif]

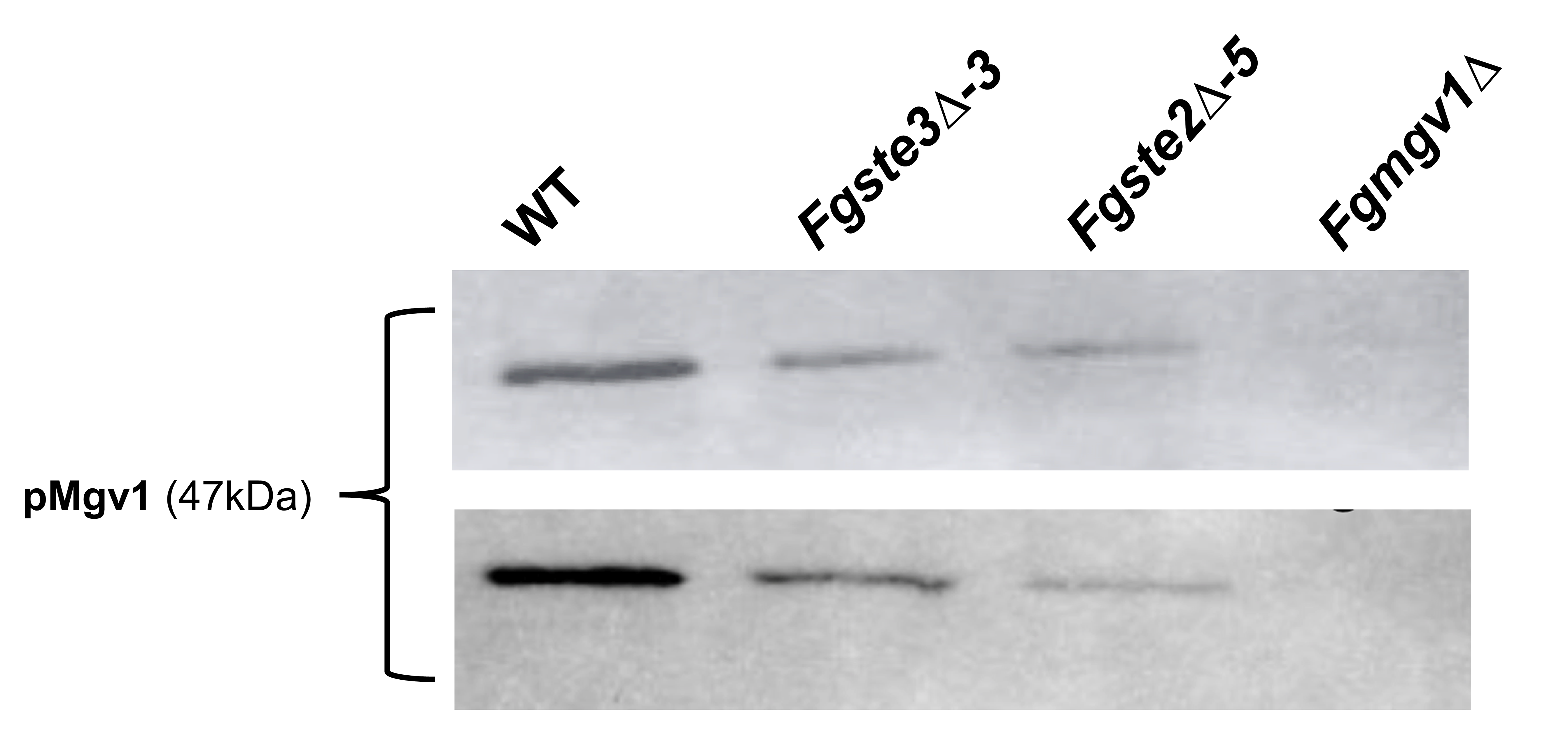

Supplement: FIG S5 [file msphere.00456-22-s0005.tif]

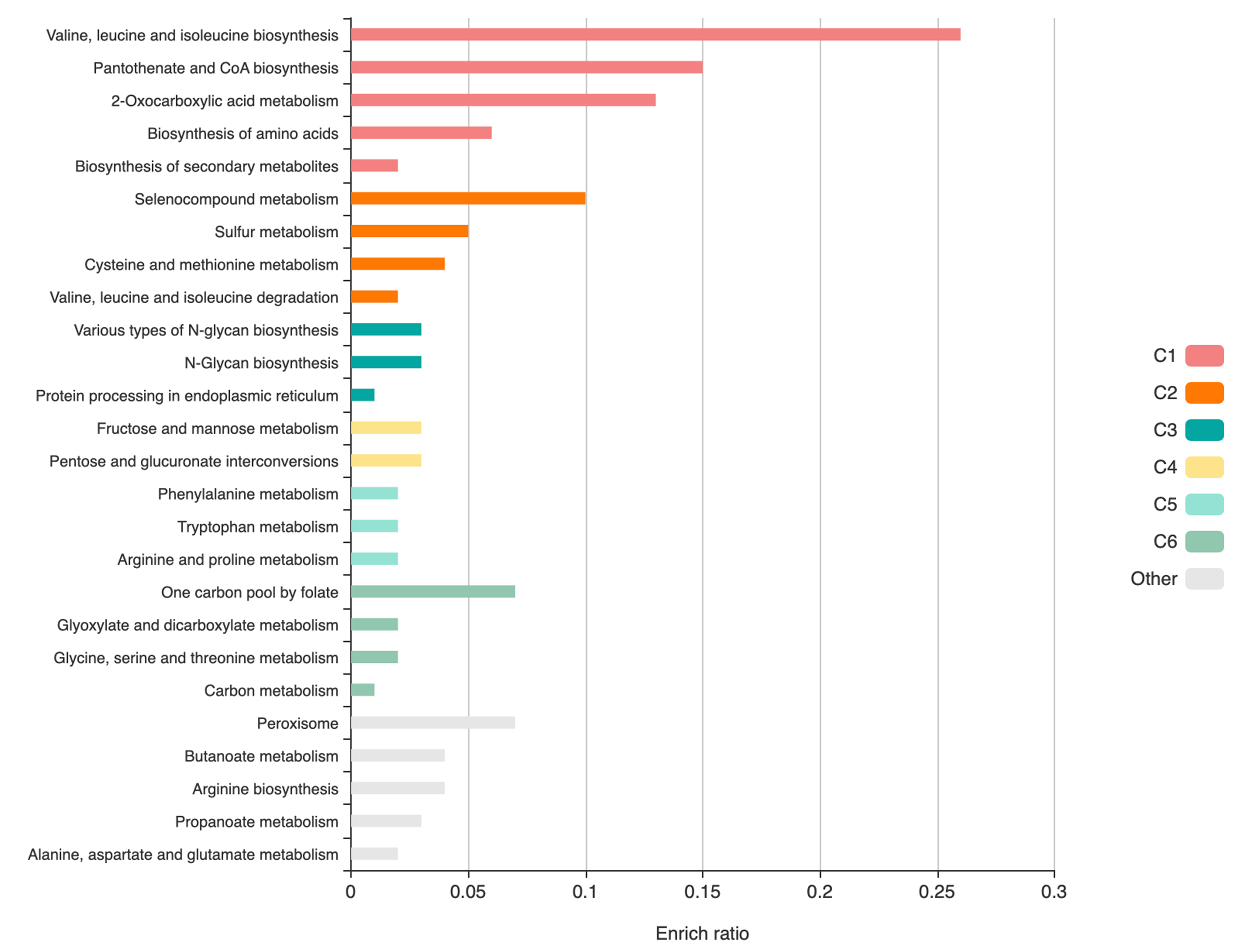

Supplement: FIG S6 [file msphere.00456-22-s0006.tif]
